# Seroprevalence of Rift Valley and Crimean-Congo Hemorrhagic Fever Viruses, Benin, 2022–2023

**DOI:** 10.3201/eid3108.250020

**Published:** 2025-08

**Authors:** Nadine Olk, Anges Yadouleton, Olga Quenum, Stephane Sohou, Aime Goundote, Grace Aho Glele Rodrigue, Blaise Guezo-Mevo, Sonia Bedie, Michael Nagel, Petra Emmerich, Benjamin Hounkpatin, Jan Felix Drexler

**Affiliations:** Charité-Universitätsmedizin Berlin, corporate member of Freie Universität Berlin, Humboldt-Universität of Berlin, Institute of Virology, Berlin, Germany (N. Olk, J.F. Drexler); Ecole Normale Supérieure de Natitingou, Université Nationale des Sciences, Technologies, Ingénierie et Mathématiques, Cotonou, Benin (A. Yadouleton); Laboratoire des Fièvres Hémorragiques Virales, Ministry of Health, Cotonou (A. Yadouleton, O. Quenum, S. Sohou, A. Goundote, G.A.G. Rodrique, B. Guezo-Mevo, S. Bedie, B. Hounkpatin); Deutsche Gesellschaft für Internationale Zusammenarbeit, Bonn, Germany (M. Nagel); Bernhard Nocht Institute of Tropical Medicine, Hamburg, Germany (P. Emmerich); Center of Internal Medicine, University Medicine Rostock, Rostock, Germany (P. Emmerich); German Centre for Infection Research, associated partner Charité-Universitätsmedizin, Berlin (J.F. Drexler)

**Keywords:** arbovirus, viruses, Rift Valley fever, Crimean-Congo hemorrhagic fever, zoonoses, fever of unknown origin, Benin

## Abstract

We screened 650 febrile patients from Benin for Rift Valley fever and Crimean-Congo hemorrhagic fever viruses during 2022–2023. None were positive by reverse transcription PCR; 1.1% and 0.3%, respectively, had virus-specific IgG. False-positive results from malaria-associated antibodies likely reacting with histidine-tagged viral antigens mandate careful validation of serologic tests in malaria-endemic regions.

Rift Valley fever virus (RVFV; family Phenuiviridae) and Crimean-Congo hemorrhagic fever virus (CCHFV; family Nairoviridae) are arthropodborne viruses endemic to Africa and the Arabian Peninsula (*1*,*2*) and high-priority pathogens that can cause lethal hemorrhagic fever (*2*–*4*) (https://www.who.int/publications/m/item/WHO-BS-2023-2449). In West Africa, RVFV and CCHFV are considered endemic in Senegal and Mauritania (*1*,*2*); regional circulation seems likely in Guinea, Burkina Faso, Ghana, and Nigeria (*2*). In Benin, CCHFV antibodies were reported in humans in 1981, but RVFV and CCHFV epidemiology remains unknown (*1*,*2*). Both RVFV and CCHFV infect diverse animals reared as livestock (*3*). Benin has been undergoing changes in traditional cattle farming, including increased herd sizes and sedentarization (*5*), which may intensify RVFV and CCHFV circulation. We collected serum samples for routine diagnostic examinations for RVFV and CCHFV in 7 hospitals located across ≈700 km and 3 ecozones in Benin (Appendix Table, Figure).

We investigated serum samples from 650 febrile patients (mean age 26.7 [interquartile range 18–34] years; 70.3% female, 29.7% male) who were seen during December 2022–January 2023. We analyzed samples for acute RVFV and CCHFV infection using PCR-based methods and had no positive results (Appendix). However, we detected IgG by using commercially available ELISA kits (RVFV, competitive ELISA; ID.Vet, https://bioadvance.life/en/id-vet-2; CCHFV, indirect ELISA; Euroimmun, https://www.euroimmun.com) with viral nucleoproteins as antigens. We confirmed CCHFV ELISA results by using a CCHFV immune complex capture IgG ELISA (Panadea Diagnostics, https://www.panadea-diagnostics.com) and RVFV and CCHFV ELISA results by indirect IgG immunofluorescence assays (IFAs).

The competitive RVFV IgG ELISA was positive in 10 (1.5%, 95% CI 0.6%–2.5%) samples; 7 were positive by RVFV IFA with high endpoint titers of 1:1,000–12,500 serum dilution (Figure 1, panel A; Figure 2, panel A; Appendix Table). Differential test sensitivity might cause discordant ELISA and IFA results, but ELISA reactivity was not weaker in IFA-negative samples compared with IFA-positive samples (p = 0.83 by Mann-Whitney U test). By indirect ELISA, 40 (6.1%, 95% CI 4.3%–8.0%) samples were positive for CCHFV, but only 5 samples tested positive by immune capture ELISA (Figure 2, panels B, C). Of those 5 samples, we confirmed 2 by CCHFV IFA, with low endpoint titers of 1:20–1:80 (Figure 1, panel B; Appendix Table). IFA-negative samples showed low reactivity in the immune capture ELISA, suggesting differential sensitivity or need to adjust ELISA positivity thresholds (Figure 2, panel C). 

**Figure 1:**
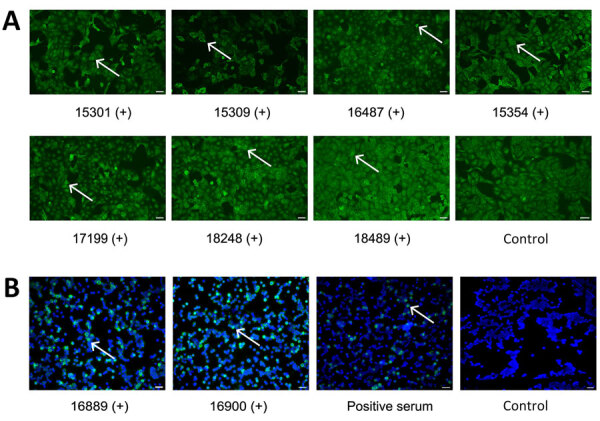
IFA for IgG against Rift Valley and Crimean-Congo hemorrhagic fever viruses, Benin, 2022–2023. A) Serum samples were tested using a commercial IFA (Euroimmun, https://www.euroimmun.com) with Rift Valley fever virus–infected Vero cells. Positive samples are shown at 1:100 dilution; white arrows mark infected cells. B) Serum samples were tested using in-house IFA with Crimean-Congo hemorrhagic fever virus–infected Vero cells (Appendix). Positive samples are shown at 1:10 dilution; white arrows mark infected cells. Titers are provided for the individual samples (Appendix Table). Noninfected controls are shown. Scale bars indicate 20 μm. +, positive serum sample; IFA, immunofluorescence assay.

**Figure 2:**
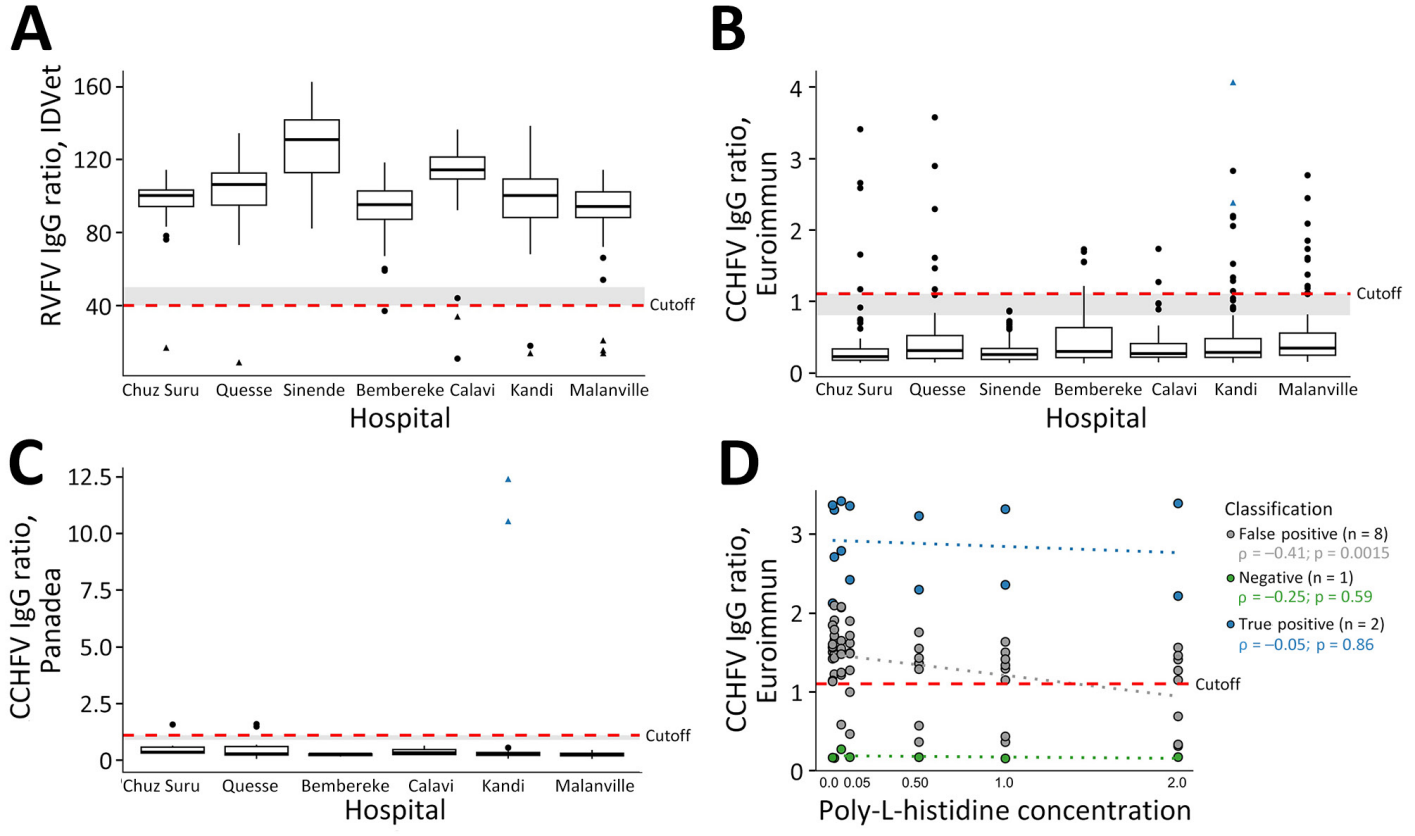
ELISA reactivity for Rift Valley and Crimean-Congo hemorrhagic fever viruses, Benin, 2022–2023. IgG ELISA (ID.Vet, https://bioadvance.life/en/id-vet-2) for RVFV for which a sample/negative percentage <40.0 is considered positive. IgG ELISAs (Euroimmun, https://www.euroimmun.com; Panadea Diagnostics, https://www.panadea-diagnostics.com) for CCHFV for which ratios >1.1 are considered positive according to the manufacturer. A) RVFV competitive ELISA (ID.Vet) using nucleoprotein as antigen. Positive samples, n = 10/650. B) CCHFV indirect ELISA (Euroimmun) using nucleoprotein as antigen. Positive samples, n = 40/650. C) CCHFV immune complex capture ELISA (Panadea) using nucleoprotein as antigen. Positive samples, n = 5/92. D) Reduced indirect IgG ELISA reactivity of CCHFV (Euroimmun) with poly-L-histidine concentrations of 0.01, 0.05, 0.10, 0.50, 1.00, and 2.00 mg/mL. Box plots show sample distribution, displaying medians (thick lines within boxes) and interquartile ranges (box top and bottom edges); whiskers indicate 1.5× interquartile range. Red lines show cutoff levels for ELISAs; gray shading shows the area for borderline results; black triangles show samples positive by RVFV immunofluorescence assay; blue triangles show samples positive by CCHFV immunofluorescence assay. The Spearman correlation was performed in R, and boxplots for RVFV and CCHFV were plotted using the ggplot2 package in R (https://www.r-project.org). Because of the low detection rates of RVFV-specific and CCHFV-specific IgG, negative reverse transcription PCR results, and low serum volumes, we did not perform IgM analyses. CCHFV, Crimean-Congo hemorrhagic fever virus; RVFV, Rift Valley fever virus.

Discrepancies among detection rates of the 2 CCHFV ELISA tests and IFA were surprising. Unspecific ELISA reactivity can occur; for example, malaria or herpes virus infection might cause unspecific B-cell stimulation (*6*,*7*). Antibodies against *Plasmodium falciparum* parasites’ histidine-rich proteins occur in ≈25% of people in malaria-endemic areas and decrease sensitivity of rapid diagnostic tests (*8*). During in vitro antigen production for serologic tests, <6 histidine residues are frequently added to expression constructs for protein purification (*9*). Increasing externally added histidine concentrations led to significantly decreased CCHFV indirect ELISA reactivity in potentially false-positive samples (ρ = 0.41; p = 0.0015) (Figure 2, panel D). In contrast, reactivities of likely true-positive samples (i.e., confirmed by IFA or immune capture ELISA) and likely true-negative samples were not affected by incremental histidine concentrations. Those data substantiated that antibodies potentially elicited by previous or acute *Plasmodium* infections targeting histidine-rich epitopes might have interacted with likely histidine-tagged indirect ELISA antigens to cause the observed reactivity pattern, including multiple likely false-positive test results. Other histidine-rich immunogens might also have elicited potentially cross-reactive antibodies, yet malaria-associated immune responses remain the most plausible explanation because of the abundance of malaria in sub-Saharan Africa and a similar rate of potentially false-positive COVID-19 results in a previous serologic study (*6*). Although the competitive RVFV ELISA and immune complex capture–based CCHFV ELISA might be more specific than indirect ELISA formats, we only considered IFA-positive results for a conservative assessment of the RVFV antibody detection rate of 1.1% (95% CI 0.3%–1.9%; n = 7/650) and of the CCHFV antibody detection rate of 0.3% (95% CI −0.1% to 0.7%; n = 2/650) (Appendix Table). 

Our serologic data thus substantiated circulation of RVFV and CCHFV in Benin (Appendix Table), albeit at relatively low rates that are largely comparable to neighboring countries (*1*,*2*). Livestock rearing in Benin is transitioning to partly sedentary systems with larger cattle herds (*5*), which highlights the need to continuously monitor RVFV and CCHFV circulation in humans and cattle (*10*) and support with robust serologic tests validated for specificity in malaria-endemic regions and direct detection of pathogens in arthropod vectors, such as *Culex* and *Aedes* mosquitoes for RVFV and *Hyalomma* ticks for CCHFV.

The main limitation of our study is that it is a nonrepresentative sample. However, including febrile patients from 7 hospitals across 3 ecozones provides broad geographic and ecologic coverage (*1*).

Beyond surveillance, strategies for future vaccination of livestock and humans will benefit from robust epidemiologic data on RVFV and CCHFV to efficiently use resources across sub-Saharan Africa. Serologic tests relying on tag-free protein production, alternative tags, and careful validation of histidine-tagged antigens for specificity are mandatory for use of antibody tests in malaria-endemic regions.

Appendix Additional information for seroprevalence of Rift Valley and Crimean-Congo hemorrhagic fever viruses, Benin, 2022–2023.
