# Supplementary material for: Seroprevalence of Rift Valley and Crimean-Congo Hemorrhagic Fever Viruses, Benin, 2022–2023
Source: Emerg Infect Dis. 2025 Aug;31(8):1662–5. doi: 10.3201/eid3108.250020 (PMC12309760; doi:10.3201/eid3108.250020)
Supplement: Appendix — Additional information for seroprevalence of Rift Valley and Crimean-Congo hemorrhagic fever viruses, Benin, 2022–2023. [file 25-0020-Techapp-s1.pdf]

Article DOI: https://doi.org/10.3201/eid3108.250020

EID cannot ensure accessibility for supplementary materials supplied by authors. Readers who have difficulty accessing supplementary content should contact the authors for assistance.

Seroprevalence of Rift Valley and Crimean-Congo Hemorrhagic Fever Viruses, Benin, 2022–2023

Appendix

Material and Methods

Geographic presentation of Rift Valley fever virus (RVFV) and Crimean-Congo Hemorrhagic fever virus (CCHFV) in Benin

The map of Benin (red) in Africa was generated by using the Natural Earth package in R (https://www.r-project.org). Sampling sites within Benin and geographic distribution of RVFV- and CCHFV-specific IgG antibodies among 7 hospitals in three ecozones of Benin are shown within the map of Benin. For the statistical analysis (Appendix Table), the following ecozones were combined: the Guinea-Congolian (light blue) and Coastal (gray-blue) zones were merged into the Guinea-Congolian zone; the North Sudanian (dark gray) and South Sudanian (light gray) zones were grouped as the Sudanian zone; and the North Guinea (blue) and South Guinea (dark blue) zones were combined into the Guinea zone. Benin global administrative boundary data were obtained from GADM database (Global Administrative Areas Database).

Cohort description among ecozones and hospitals in Benin

Serum samples from febrile patients (n = 650), collected between December 2022 and January 2023 in seven hospitals across ≈700 km of longitude in the Guinea-Congolian, Guinean, and Sudanian ecozones of Benin, were tested for RVFV and CCHFV. Missing data were coded as NA and excluded from all analyses using complete case analysis. Statistical description and analysis were performed with the tidyverse and rstatix package in R (https://www.r-project.org). The study was conducted in accordance with the STROBE (Strengthening the Reporting of Observational Studies in Epidemiology) guidelines.

Appendix Table. Description of febrile patient characteristics sampled in seven hospitals in Benin*

Hospital	No. of samples	Mean age, y, (95% CI)	% F/% M	RVFV IFA IgG positive, no. (95% CI)	Sample no. (RVFV IgG positive)	RVFV IgG IFA,titer (n = 7/10)	CCHFV IFA IgG postive, no. (95% CI)	Sample no. (CCHFV IgG positive)	CCHFV IgG IFA, titer (n = 2/5)
Guinea-Congolian zone, n = 158		30 (26–34)	66/34	2 (–0.5 to 3.0)			0 (0−0)		
HZ CHUZ SURU-LERE	80				18498	1:5.000		—	—
HZ CALAVI	78				16487	1:1.000			
					—	—			
Guinea zone, n = 80		31 (28–34)	75/25	1 (–1.2 to 3.7)			0 (0–0)	—	—
CS QUESSE	80				18248	1:5.000		—	—
Sudanian zone, n = 412		24 (23–26)	71/29	4 (0.0–1.9)			2 (–0.2 to 1.2)		
CS SINENDE	84				—	—			
HZ KANDI	161				17199			16889	1:20
						1:5.000		16900	1:80
HZ MALANVILLE	84				15301	1:1.000			
					15309	1:12.500			
					15354	1:10.000			
HZ BEMBEREKE	83				—	—			

*CCHFV, Crimean-Congo hemorrhagic fever virus; IFA, immunofluorescence assay; RVFV, Rift Valley fever virus.

Molecular detection

Screening for phlebovirus RNA was performed by broadly reactive nested RT-PCR, using a previously published test (1). Detection of all currently known Crimean-Congo hemorrhagic fever virus (CCHFV) genotypes was performed by one-step multiplex real-time RT-PCR as published (*2*).

Competitive ELISA for Rift Valley Fever (RVFV) antibodies (IgG), ID.Vet

The samples, positive and negative controls were diluted in a 1:1 ratio with the dilution buffer. 100 µl of the positive control, negative controls, and diluted patient samples were transferred into the respective microplates. The samples were incubated for 60 minutes at 37°C±2°C. The microplate was washed three times, with 300 µL of washing buffer. 100 µL of the prediluted conjugate was pipetted into each of the microplate wells and incubated for 30 minutes at room temperature. The microplate was then washed three times as described above. 100 µl of substrate solution was pipetted and the microplates were incubated protected from light for 15 minutes at room temperature. Then, 100 µl of stop solution was pipetted into each well. The color intensity was measured photometrically at a wavelength of 450 nm and a reference wavelength between 620 and 650 nm within 30 minutes of adding the stop solution.

Calculation of the competitive-RVFV IgG Elisa results

The extinction value of the negative control establishes the lower limit of the reference range for non-infected individuals (cutoff), as recommended by the manufacturer. Values below the specified cutoff are considered positive, while those higher the cutoff are considered negative. The ratio is calculated according to the following formula:

$S_{ample}/N_{egative}\%$ = (Extinction of the control or patient sample)/(Extinction of negative control) *100

The ratio results were interpreted as follows:

S/N% >50%: negative

S/N% <S/N% ≥50%: Questionable

S/N% ≤40%: positive

Indirect IgG ELISA for CCHFV, Euroimmun

The samples, positive and negative controls were diluted in a 1:100 ratio with the dilution buffer. 100 µl of the calibrator, positive control, negative controls, and diluted patient

samples were transferred into the respective microplates. The samples were incubated for 60 minutes at 37°C±1°C. The microplate was washed three times, with 300 µL of washing buffer. 100 µL of the conjugate was pipetted into each of the microplate wells and incubated for 30 minutes at room temperature. The microplate was then washed three times as described above. 100 µl of substrate solution was pipetted and the microplates were incubated protected from light for 15 minutes at room temperature. Then, 100 µl of stop solution was pipetted into each well. The color intensity was measured photometrically at a wavelength of 450 nm and a reference wavelength between 620 and 650 nm within 30 minutes of adding the stop solution.

Calculation of the indirect-CCHFV ELISA results

The extinction value of the calibrator establishes the upper limit of the reference range for non-infected individuals (cutoff), as recommended by the manufacturer. Values exceeding the specified cutoff are considered positive, while those below the cutoff are considered negative. The ratio is calculated according to the following formula:

Ratio = (Extinction of the control or patient sample)/(Extinction of calibrator)

The ratio results were interpreted as follows:

Ratio <0.8: Negative

Ratio ≥ 0.8 to <1.1: Borderline

Ratio ≥ 1.1: Positive

Immune complex ELISA for CCHFV antibodies (IgG), Panadea

The samples were inactivated with 2% Triton X. Serum samples, positive control and negative were diluted 1:50 with serum dilution buffer. 25µl of diluted conjugate (1:100, recombinant HRP-labeled CCHFV antigen) and 25µl of diluted samples, negative and positive control were transferred into the respective microplates. The samples were incubated for 24hr at 2°C – 8°. The microplate was washed three times, with 300 µL of washing buffer. 100 µl of substrate solution was pipetted and the microplates were incubated protected from light for 15 minutes at room temperature. Then, 100 µl of stop solution was pipetted into each well. The color intensity was measured photometrically at a wavelength of 450 nm minus 620 nm within 30 minutes of adding the stop solution. Samples are co-incubated together with a horseradish peroxidase (HRP)-labeled recombinant CCHFV antigen in a microwell plate coated with a recombinant IgG immune complex specific capture molecule (https://www.innovative-diagnostics.com).

Calculation of the immune complex-CCHFV Elisa results

The calculation of $OD_{neg,av}$ was performed from the absolute OD values of two negative controls. The cutoff value was calculated with the following formula:

$OD_{CO} = OD_{neg,an} + 0.150$

The index values for the samples were calculated according to the manufacturer:

Index (IV) = $(OD450_{sample} – OD620_{sample}) / = D_{CO}$

The ratio results were interpreted as follows:

$IV_{sample} > 1.100$: positive

$0.900 < IV_{sample} < 1.100$: Equivocal

$IV_{sample} \leq 0.900$: negative

Immunofluorescence assay for RVFV antibodies (IgG), Euroimmun

Serum samples were tested with a commercial IFA slide from EUROIMMUN AG, in which cover glasses are coated with RVFV-infected Vero cells cut into millimeter-sized fragments and fixed on a Biochip. Diluted samples were transferred onto slides with infected and non-infected cells indicated as 2 Biochips per field. Samples are characterized by distinct punctate fluorescence patterns in the cytoplasm of infected cells in the cytoplasm. This pattern reflects the presence of viral proteins cells as negative samples may still take up the stain non-specifically, but with no granular-like pattern. For better visual representation, the microscopic images of the positive samples are shown at a 1:100 dilution and an arrow pointing to positive cells in each image.

Immunofluorescence assay for CCHFV antibodies (IgG), BNITM

Serum samples were tested with an immunofluorescence assay using CCHFV virus-infected cells as antigen. The slide production requires a biosafety level 4 laboratory and was produced and provided by PD Dr. Emmerich's group at the Bernhard Nocht Institute. The cover glasses are coated with CCHFV-infected Vero cells and acetone-fixed. Diluted samples (1:10 – 1:5120) were transferred into a slide with cells infected with CCHFV. The cells were stained with goat-anti-human FITC 1:1000 antibody as a secondary antibody to detect human CCHFV-specific IgG and DAPI for nuclei staining. For better visual representation, the microscopic images of the positive samples are shown at a 1:10 dilution. Positive serum (ID K14–7B) with an IgG titer of 1:1280 was provided by PD Dr. Emmerich's group at the Bernhard Nocht Institute.

Reduced CCHFV IgG ELISA (Euroimmun) reactivity upon addition of external histidine

Serum samples, positive and negative tested with indirect CCHFV IgG ELISA, were diluted 1:100 with different concentrations of poly-L-histidine (stock 10mg/ml). A dilution series of 2 mg/ml, 1 mg/ml, 0.5 mg/ml, 0.1 mg/ml, 0.05 mg/ml and 0.01 mg/ml of poly-L-histidine (Sigma-Aldrich, Cat# P9386–10MG) was added to serum dilution and transferred to the respective microplates. ELISA was performed as previously described.

References

1. Marklewitz M, Dutari LC, Paraskevopoulou S, Page RA, Loaiza JR, Junglen S. Diverse novel phleboviruses in sandflies from the Panama Canal area, Central Panama. J Gen Virol. 2019;100:938–49. PubMed https://doi.org/10.1099/jgv.0.001260

2. Sas MA, Vina-Rodriguez A, Mertens M, Eiden M, Emmerich P, Chaintoutis SC, et al. A one-step multiplex real-time RT-PCR for the universal detection of all currently known CCHFV genotypes. J Virol Methods. 2018;255:38–43. PubMed https://doi.org/10.1016/j.jviromet.2018.01.013

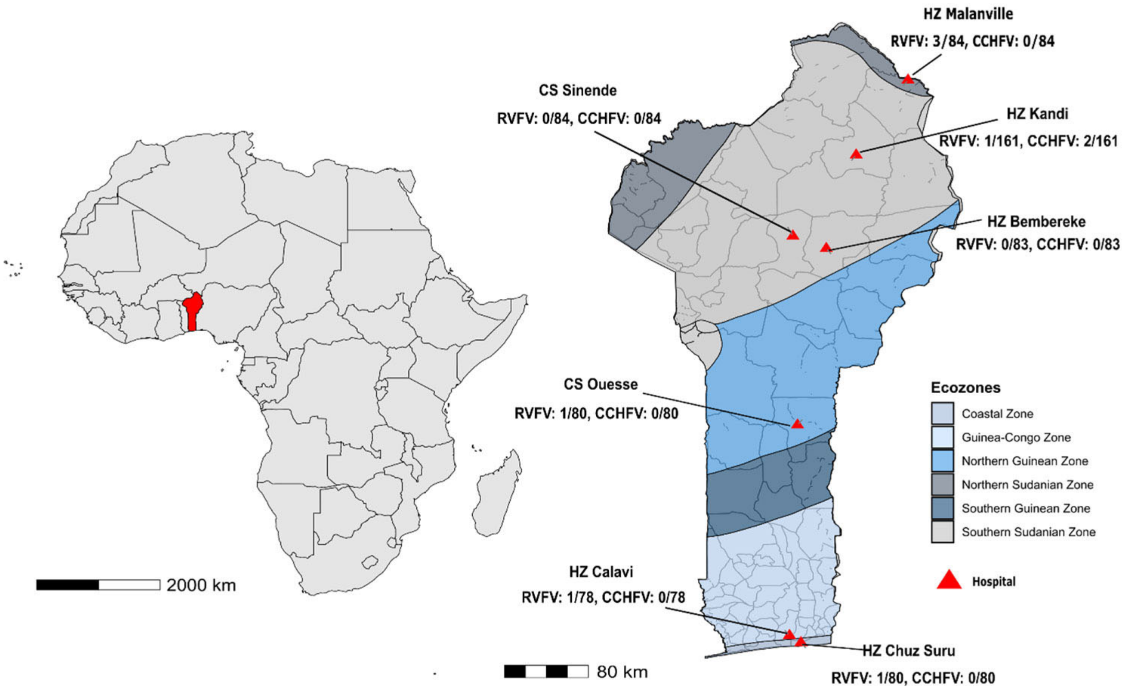

Appendix Figure. RVFV-specific and CCHFV-specific IgG antibody detection in Benin, December 2022–January 2023.
